# Single Step Synthesis and Functionalization of Nano Titania for Development of Multifunctional Cotton Fabrics

**DOI:** 10.3390/ma15010038

**Published:** 2021-12-21

**Authors:** Faiza Safdar, Amjed Javid, Munir Ashraf

**Affiliations:** Functional Textile Research Group, National Textile University, Faisalabad 37610, Pakistan; faizatch@gmail.com (F.S.); amjad1238@gmail.com (A.J.)

**Keywords:** antibacterial, cotton, multifunctional, superhydrophobic, UV protection

## Abstract

Synthesis and modification of nanoparticles to make them suitable to functionalise a substrate for various application fields involves many steps, which are complex, time-consuming, and sometimes require special equipment. This is a major drawback to meet rapid technological requirements. In this work, a procedure has been developed to modify TiO_2_ nanoparticles by the sol-gel method at their synthesis stage using titanium tetraisopropoxide and modifying agents including ODS and GPTMS. The prepared nanoparticle finish can be used as it is without any further processing, thus eliminating the need for extra steps required to decorate them on some substrate. The nanoparticles were characterised by SEM, EDX, FTIR, XRD, and zeta potential. The adhesion of the obtained nanoparticles was tested by applying them to a cellulosic substrate. The obtained substrate was subjected to mechanical action and adhesion efficiency was estimated on the basis of UV transmittance and antibacterial properties that showed excellent results. The hydrophobic properties of the obtained nanoparticles were assessed by measuring water contact angles, which reached 157.9°, indicating their superhydrophobic nature. The developed procedure is facile and will be suitable for the engineering of multiple surfaces.

## 1. Introduction

Surface modification of textiles by physical and chemical processes plays an important role in developing high-performance textiles. The performance capability of textiles for a particular application depends on the final characteristics of the developed material. In the past few years, researchers have been trying to develop high-performance textiles using nanomaterials and, in doing so, new properties are being imparted, resulting in the development of technical textiles. Therefore, nanotechnology is becoming vital to developing multifunctional products at a commercial level. It is worth mentioning that due to its popularity, consistency, reliability, and effectiveness, it is also gaining commercial potential for the textile industry [1]. The promising properties of nanostructured modified textile materials such as UV protection, antibacterial, and self-cleaning without significantly impairing breathability or hand feel can be achieved due to the large surface area and high surface energy of nanomaterials.

In recent years, functionalised cotton fabrics bearing self-cleaning, UV shielding, and antibacterial properties have acquired more attention due to their everyday applications in a wide range of fields [2,3,4,5]. For multifunctionality, the surface of cotton fabrics has been modified using various nano-scale materials including titania [4], silica [6], zinc oxide [7], and silver [8] to impart multifunctional characteristics. For example, titania nanoparticles have been extensively researched for their ability to impart UV protection [9], antibacterial [10], and self-cleaning [10] properties to fabrics. The popularity of titania-based nanomaterials is due to their exceptional catalytic properties under light, and outstanding features like hypoallergenicity, cost-efficiency, and chemical stability. Three recognised crystallographic phases of nano titania are rutile, anatase, and brookite. Of these, the anatase phase has been widely studied due to its outstanding photocatalytic properties [11].

Titania nanoparticles can be synthesised by various methods, such as sol-gel, hydrothermal, solvothermal, and emulsion precipitation [12,13,14,15,16]. High cost, longer reaction time, and complex processes comprising many steps are some of the limitations of some of these processes. Various important factors that may affect the particle size, crystal structure, and morphology of as-synthesized nanoparticles are solution concentration [17], reaction time [18], pH, and homogeneous dispersion of the solution [19,20]. Among various methods, the sol-gel method is the most widely adopted for the preparation of nano-titania due to its simplicity, low cost, and high yield.

In general, there are two types of sol-gel methods; one is aqueous-based and the other is non-aqueous-based. Titania nanoparticles of different morphologies can be obtained by choosing a solvent of different polarity, such as a polar or non-polar solvent [21]. The most widely adopted method for the synthesis of titanium oxide nanoparticles is the aqueous-based sol-gel method, which uses titanium alkoxides as a precursor for titanium oxide. In this system, water acts as an oxygen donor for the hydrolysis of the precursor [22]. As titanium alkoxides are highly sensitive to moisture and air, their hydrolysis in water media is spontaneous and, if not properly controlled, can lead to the formation of amorphous TiO_2_ particles, requiring subsequent heat treatment at high temperatures to impart crystallization [23,24]. On the other hand, the nonaqueous route is advantageous in terms of controlled hydrolysis and imparting crystallinity to the obtained TiO_2_ nanoparticles at the same time. The nonaqueous route is further subdivided into solvent-and surfactant-controlled synthesis techniques. In such a system, organic molecules present act as oxygen donors, resulting in the controlled hydrolysis of titania precursors. Moreover, such a process is easy to control, has a low cost, and the obtained particles are pure [25,26]. Carlucci et al. adopted the non-aqueous system to synthesise shape-controlled TiO_2_ nanocrystals using benzyl alcohol as a solvent [27].

Titania nanoparticles prepared by the sol-gel method have been employed to impart various properties to the fabrics. Galkina et al. developed titania sol by acidic hydrolysis of titanium tetraisopropoxide in isopropanol solvent to apply to cotton fabrics already coated with 1, 2, 3, 4-butanetetracarboxylic acid (BTCA) solution, which acted as a binding agent between TiO_2_ NPs and the cellulosic chains of cotton [28]. However, the method for the modification of cotton fabric was complex and time-consuming, involving multiple steps.

Along with other characteristics, the hydrophobic properties of cotton fabrics are becoming one of the essential properties of fabrics. The synergistic effect of the surface chemical composition and surface topography reveals the superhydrophobicity. In two ways, nano-roughness to alter surface topography can be generated in two ways, viz. using nanoparticles and by several processing methods such as the sol-gel method [29], dip coating [30], the layer-by-layer technique [31], chemical vapour deposition [32], electrospinning [33], and so on. The former method comprises the preparation of nanoparticles that is complex and time-consuming as many steps are involved. Moreover, the limitations also include the agglomeration of particles, which hinder uniform dispersion and requires special equipment [34]. However, some of the later processing techniques also need special equipment.

Most of the strategies to develop fabrics exhibiting antibacterial and UV shielding properties along with superhydrophobicity comprise two-stage processes [35]. The first stage comprises decorating the fabric surface with nanoparticles to generate nano roughness that is accompanied by lowering the surface energy of the fabric by treating it with some hydrophobic agent [35,36,37,38]. Another way is using as-synthesized nano titania and its modifications, which have the problem of agglomeration. In the first step, the agglomeration of titania nanoparticles is broken using special equipment like sonication for a longer time to prepare their uniform dispersion, and in the second step, their modification is done with a hydrophobic moiety. Such hydrophobic titania nanoparticles cannot be applied to a substrate as it is and need further steps to make them suitable for application on a substrate [39]. This method also has a disadvantage as it requires special equipment like a sonicator, which is required to break agglomerates of nanoparticles powder and has the severe limitation of localised heating, causing a temperature rise that is not required in some cases. Another drawback is that many steps are involved, making the whole process complex and time-consuming. Hence, the development of multifunctional fabrics containing superhydrophobicity, UV protection, and antibacterial properties in a single stage, avoiding all the above-mentioned limitations, was a challenge that has been met in the present work. This will lead to the development of a one-stage process, thereby minimizing the time and cost as well. There is no such report in the literature that addresses the modification of titanium nanoparticles at their synthesis stage in a single bath using Octadecyltrimethoxysilane (ODS) and (3-Glycidoxypropyl) trimethoxy-silane (GPTMS) to prepare a finish that can be used as it is without special equipment, high temperature conditions, or a long treatment time of the fabric. GPTMS and ODS were selected as modifying agents. GPTMS is a versatile silane coupling agent having the capability to maintain the dispersion stability of nanoparticles and provide covalent links between the nanoparticles and the substrate [40]. ODS is a fluorine-free hydrophobic agent that is used to reduce the surface energy of a material in order to render it hydrophobic properties [39]. The whole procedure was based on the sol-gel method. SEM, EDX, FTIR, zeta potential analyser, and XRD were used to characterise the prepared particles. Due to the presence of epoxy and siloxane groups, the prepared finish can be applied to substrates carrying functional groups such as amine, hydroxyl, and amide, leading to the establishment of covalent graft amalgamation under certain conditions. For instance, the prepared finish was applied to a cellulosic substrate by the industrial pad-dry-cure method on conventional machinery. The surface roughness changes of the substrate were measured using the Kawabata Evaluation System. Adherence of the nanoparticles was evaluated after subjecting the treated substrate to various washing cycles and evaluating the functional properties like UV transmittance, antibacterial and hydrophobic properties after washing.

## 2. Experimental

### 2.1. Materials

For this study, plain woven cotton fabric was used in this application of synthesised sol. Titanium isopropoxide (TIP), ODS, and GPTMS (>98% reagent grade) were obtained from Sigma-Aldrich, St. Louis, MO, USA and used without further purification. Ethanol was procured from Sigma-Aldrich and double distillation was performed before use in the synthesis process. Sodium hydroxide and sodium chloride (lab grade) were purchased from Riedelde Haen, Seelze, Germany. The nutrient broth was obtained from Lab M Limited, Heywood, UK, and the nutrient agar was obtained from Merck. Two bacterial strains (*Escherichia coli* and *Staphylococcus aureus*) were kindly issued by the UAF, Pakistan.

### 2.2. Synthesis of Modified Nano Titania

A nanocomposite film was prepared on cotton fabric using the pad-dry-cure method. The finish was produced in one bath via co-hydrolysis and polycondensation reactions of ODS, TIP, and GPTMS mixtures. To develop functionalized nano titania, a 1.5% TIP solution containing ethanol and HCl was prepared, maintaining the pH of the solution at 3. The solution was set to reflux for 6 h at 75 °C to which GPTMS in different volumes was added after 2 h and ODS was added in diverse volumes mentioned in Table 1 after 1 h of the addition of GPTMS. The statistical analysis tool, Minitab^®^ 17, was used for the full factorial design of experiments using 2 factors, 3 levels of GPTMS, and 2 levels of ODS (Table 1). As the reaction proceeded, the milky solution was formed, indicating the formation of nano titania. The addition of GPTMS and ODS to the solution resulted in the grafting of these two chemicals onto the surface of nano titania. For the sake of comparison, 2% ODS was used to synthesise ODS grafted nano titania by the same method as described earlier without adding GPTMS as a control sample.

### 2.3. Preparation of Multifaceted Cotton Fabric

Before coating, the cotton fabrics were dried at 100 °C for 2 min. after treatment with an aqueous solution of NaOH to make them slightly alkaline and ready for the application of functionalised nano titania sol. Cotton fabric to be coated with ODS functionalised nano titania was dried at 100 °C for 1 min to remove any moisture present in the fabric. Then the fabrics were immersed for 1 min with continuous stirring in nano-sol. The nanoparticle solution was evenly coated by using a laboratory padding mangle machine. After padding, the cotton fabric was dried at 100 °C for 1 min. This process was repeated three times for even coating of the fabric, washed with deionized water to remove surface adhered nano titania, dried at 100 °C for 2 min, and cured at 150 °C for 3 min using a lab-scale stenter.

## 3. Testing and Characterization

The surface morphology of cotton fabrics coated with functionalized nano titania was examined by scanning electron microscopy (FEI Nova NanoSEM 450, Hillsboro, OR, USA ) at an accelerating voltage of 15 kV. To obtain clear SEM images, a sample was sputter-coated with a very fine layer of Au for 60 s. The elemental distribution on the coated textile surface was analysed by energy dispersive spectroscopy (EDS) attached to SEM.

The chemical composition of cotton fabrics before and after the coating of modified nano titania was analysed by FT-IR spectrometry (Bruker Tensor 27, Bruker Optik, Ettlingen, Germany) in attenuated total reflectance (ATR) mode in the range of 4000–400 cm^−1^.

The Bruker D2-Phaser with CuKα radiation at a wavelength of 0.154 nm was used for crystal structure analysis of functionalized nano titania.

Zetasizer Nano (Malvern ZEM-3600, Malvern Instruments Ltd. now a part of Malvern Panalytical, Malvern, UK) was employed for the determination of the surface charge present on modified nano titania.

Water contact angle was measured by the Theta Lite Optical Tensiometer (Biolin Scientific, Gothenburg, Sweden) droplet under ambient conditions. The water contact angle was observed at five different locations to calculate the average value.

The fabrics coated with modified nano titania were evaluated for their UV protection properties using the UV transmittance analyser, Labsphere, North Sutton, NH, USA. The AATCC 183-2004 standard test method was used, and the mean UPF value was automatically calculated by the instrument.

The antibacterial properties of the fabric coated with modified nano titania were evaluated quantitatively according to ISO 20743:2013 Transfer Method. The control (pristine cotton fabric) and the coated fabric were placed on the agar plates (nutrient agar), which were previously inoculated with 1 mL of inoculum (bacterial culture of 2 × 10^6^ CFU/mL) and the fabrics were pressed down by placing 200 g of weight on the fabric for 60 s. Six samples of all the specimens to be tested (control, coated unwashed, and coated fabric after 20 laundering cycles) were taken, three of which were used immediately after bacterial transfer, while the other three were used for incubation. Three samples of each tested specimen were removed from the agar surface and placed in a petri dish with the transfer surface (the surface which was in contact with the agar) facing up, and the plates were then incubated in an incubator at 37 °C for 24 h. The other triplicate set of each specimen was immediately transferred to three separate glass vials containing 20 mL of neutralizing solution, and the vials were shaken for 10–15 min. Then, six serial dilutions of the neutralizing solution were prepared by adding 1 mL of solution to a test tube previously containing 9 mL of broth (nutrient broth), and plating of dilutions was performed on agar plates. After incubation, the same procedure was repeated and bacterial counts at 0 h and 24 hrs were obtained and antibacterial activity (A) as log reduction was calculated using Equation (1).

(1) A=F−G

where F = (log Ct − logC0) and G = (log Tt − log T0), C0 and Ct are the bacterial counts of control fabric at 0 h and after 24 h, respectively, while T0 and Tt are the bacterial counts of the coated sample at 0 h and after 24 h, respectively, while A is the antibacterial activity value. The percentage reduction was calculated according to Equation (2).


(2) %agereduction=[(log Ct−log Tt)]/logCt ×100

As textiles are subjected to various washing cycles during their life, therefore, the developed fabrics were washed for various cycles according to the ISO 105 C03 method, and functional properties were tested after washing.

The tear strength of treated and untreated fabrics was determined according to the standard test method BS EN ISO 13937.

Air permeability of unmodified cotton fabric and after modification with functionalized nano titania was determined according to the AATCC 737 standard method by M021A, SDL ATLAS.

To determine the change in surface topography of fabrics treated with modified nano titania, the Kawabata evaluation system (KATO TECH Co., Ltd., Kyoto, Japan) was used.

Before performing each test, conditioning of the fabrics was done according to ISO 139:2005 at 20 ± 2 °C and 65 ± 2% relative humidity.

## 4. Results and Discussion

### 4.1. Scanning Electron Microscopy

**Figure 1:**
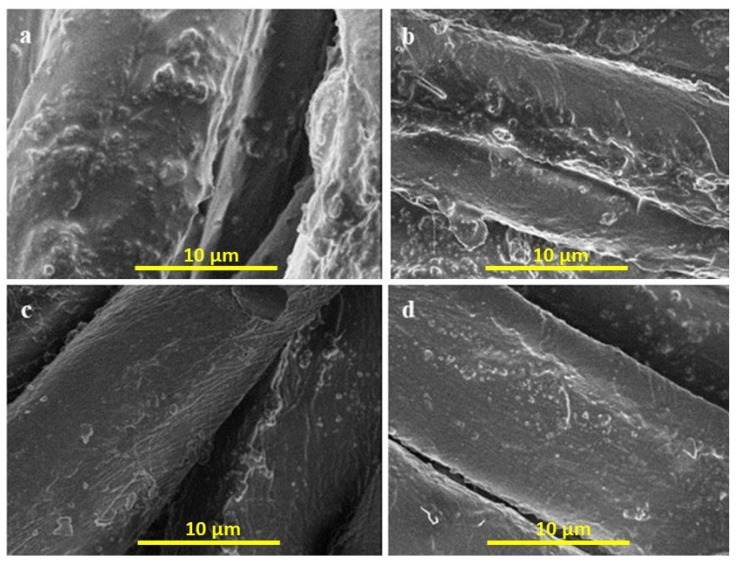
SEM images of coated cotton fabrics (**a**) unwashed sample (**b**) after 5 washing cycles. (**c**) after 10 washing cycles (**d**) after 20 washing cycles.

The surface morphology of modified nano-titania-treated cotton fabrics before and after various washings was observed by SEM. As can be observed from Figure 1, silanes along with nano titania covered the yarn. The particles are randomly distributed all over the yarns of the fabrics. Furthermore, it can be seen that NPs deposited on the surface of yarns created roughness that leads to superhydrophobicity of the fabrics. It is worth noting that after 5, 10, and 20 washing cycles, the modified nano titania are still attached to the fibre, indicating they are an integral part of the fibre. This washing durability of functionalized nano titania is predominantly due to the chemical attachment of the epoxy group of GPTMS functionalized nano titania with the hydroxyl groups of cellulose. The possibility of reaction of ODS molecules with the hydroxyl groups of cellulose cannot be ignored, as the silanol groups of organofunctional tralkoxysilanes can react not only with each other during the condensation reaction to form a continuous sol-gel matrix, but also with the hydroxyl groups on the surface of the cellulose fibres. The unwashed sample is covered with functionalized nano titania (Figure 1a) that was slightly removed after five washing cycles (Figure 1b), while the surface morphology of the fibres was not changed. Even after 10 and 20 washing cycles (Figure 1c,d, respectively), the presence of a significant amount of nano titania on the fabric surface indicates excellent washing durability of modified nano titania on the fabric surface.

**Figure 2:**
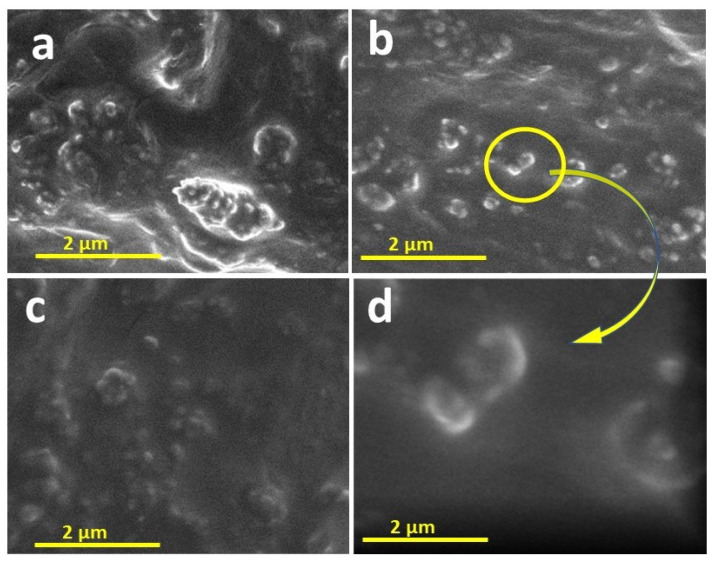
SEM images at higher magnification for (**a**) Unwashed fabric, after (**b**) 10 washing cycles, (**c**) 20 washing cycles, (**d**) higher magnification of (**b**).

Figure 2 shows the SEM images of the same treated cotton fabric at higher magnification just to elaborate on the size and attachment of NPs with fibres. Even after multiple washes, the nano titania remained firmly attached to the surface of the fibre due to the reaction of GPTMS with cellulose. Figure 2d, which is a much higher magnification of Figure 2b, shows the synthesis of functional titania in the nano range.

**Figure 3:**
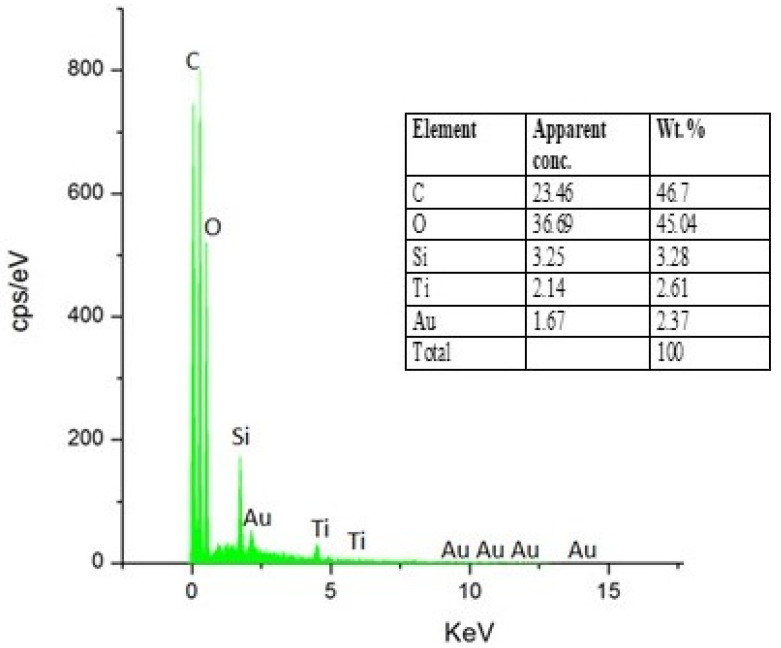
EDX spectra of cotton fabric treated with functionalized nano titania.

Furthermore, the chemical composition of the fabric treated with modified nano titania was determined by SEM-EDX. The main elements present on the surface of modified nano titania treated cotton fabric are O, C, Ti, and Si, as indicated in Figure 3. The appearance of Ti peaks is attributed to TiO_2_ nanoparticles, while the appearance of Si peaks is due to silicone elements in ODS and GPTMS.

### 4.2. Crystallography

**Figure 4:**
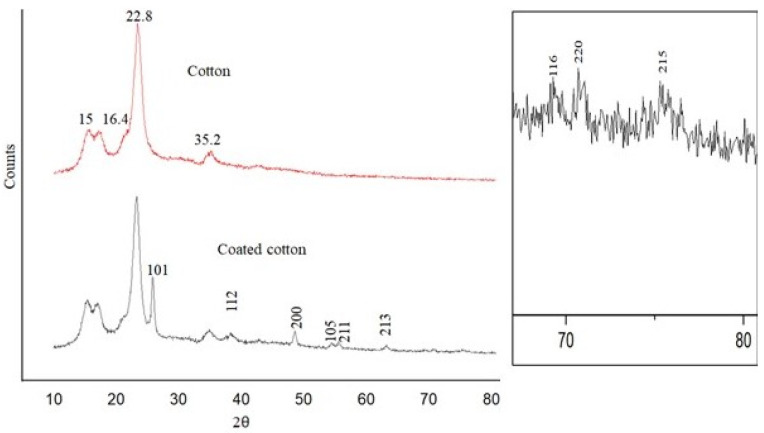
XRD pattern of synthesized modified nano titania.

An XRD pattern was measured for pristine cotton fabric and coated cotton fabric with modified nano titania in the range of 2 theta = 10–80° to identify the crystalline phase of synthesized modified nano titania as shown in Figure 4. Diffraction peaks at 15°, 16.4°, 22.8°, and 35.2° [41,42] are characteristic of native cellulose. After treatment of fabric with modified nano titania, several new peaks appeared owing to the deposition of modified nano titania. The crystalline form of synthesized modified nano titania was established through values of 2θ. For instance, the characteristic peaks positioned at ~25.7°, 39°, 48.8°, 54.8°, 60°, 63.8°, 69.9, 71.5°, and 76.4° correspond to 101, 112, 200, 105, 211, 213, 116, 220, and 215, respectively that confirms the crystalline form of synthesized modified nano titania as anatase with tetragonal geometry [43]. The characteristic peak at 23.2° represents the presence of silicates due to long chains of hydrocarbons present in silane functionalising agents [44]. The Scherrer equation (D = k λ/β cos θ) was used to calculate the particle size of synthesised modified nano titania. The full-width half maxima (FWHM) of a sharp diffraction peak at 25.7° were measured, indicating dominant crystal growth in this direction. The crystal size of synthesized modified nano titania was computed to be 17.9 nm, indicating the formation of titania at the nanoscale.

### 4.3. FTIR Analysis

**Figure 5:**
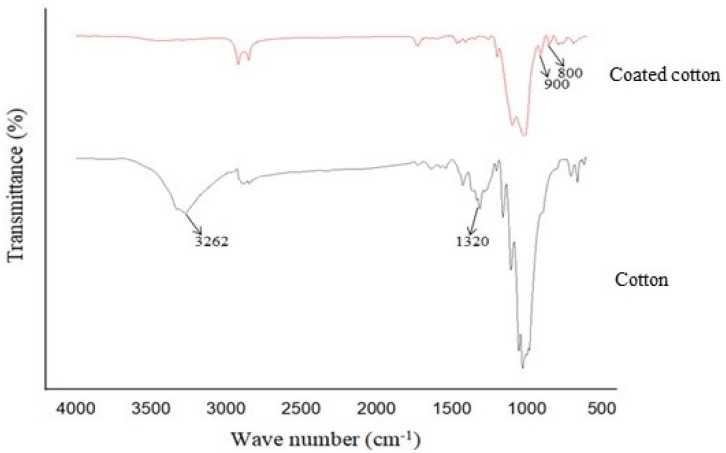
FTIR of treated cotton fabric with modified nano titania.

The chemical structure of cotton fabrics treated with modified nano titania was analysed through FTIR spectra as shown in Figure 5. It can be seen that the cotton fabric without treatment contains a broad peak of -OH within the range of 3100–3400 cm^−1^ while the same peak is missing on coated cotton fabric. This shows the evidence of crosslinking of GPTMS with cotton containing nano titania and ODS. Furthermore, GPTMS also contains a group called epoxide as a major content that reacts with cotton, and it could be that the stretching vibration of epoxide at 1225–1300 cm^−1^ is missing in the coated cotton spectrum. The peak around 790–800 cm^−1^ in coated cotton depicts the tetra-substitution-SiH bending which is available in octadecyl trimethoxysilane (ODS). The peak in the cotton fabric at 1320 cm^−1^ shows the presence of a methylol group that forms covalent bonding with other reactive groups. This peak in coated cotton is absent, showing crosslinking of GPTMS with cotton. The peak around 900 cm^−1^ in coated cotton shows the presence of silanolates, i.e., Si-O-metal (in this case, Titania) because titanium isopropoxide was applied to cotton fabric in the presence of ODS and GPTMS. This is clear evidence of the modification of nano titania with silanes.

### 4.4. Zeta Potential of Modified Nano Titania

**Figure 6:**
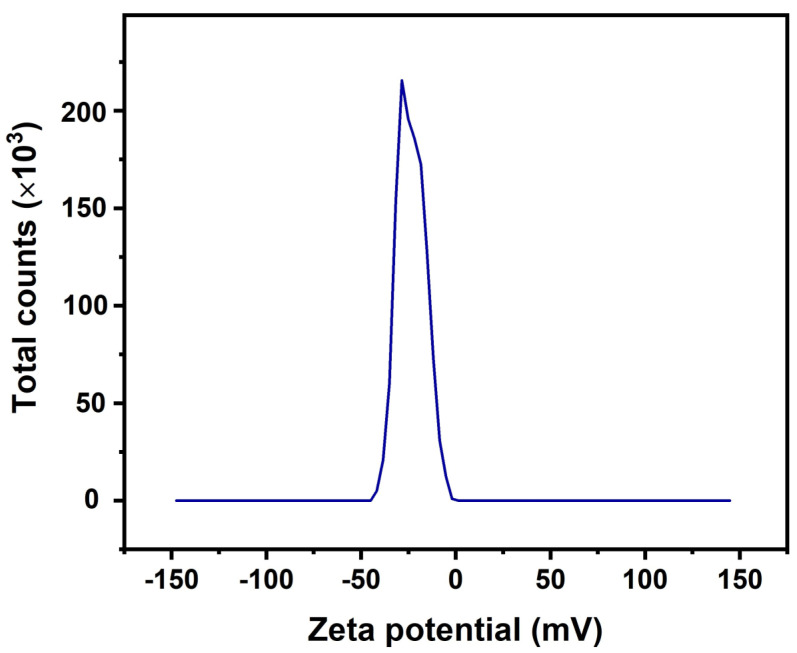
Zeta potential measurement of modified nano titania.

The surface charge carried by the prepared modified nano titania was determined by Zetasizer Nano (Malvern ZEM-3600, Malvern Instruments Ltd., now a part of Malvern Panalytical, Malvern, UK), and the results are presented in Figure 6. The apparent charge on modified nano titania was found to be −23.3 mV, which indicated good dispersion of the modified particles.

**Figure 7:**
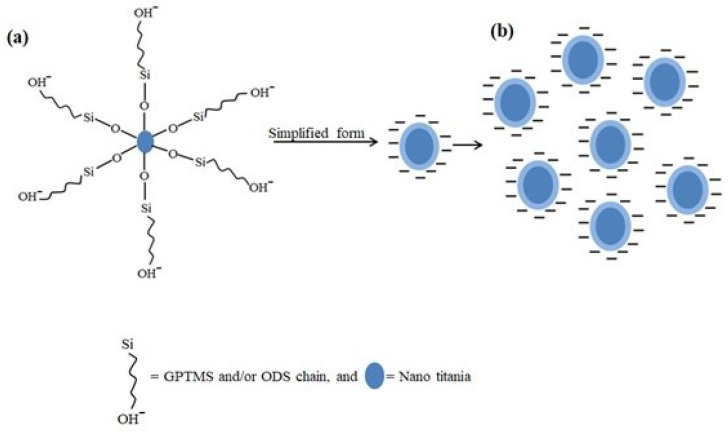
As prepared modified nano titania (**a**) in water, (**b**) Separated particles due to similar charge repulsion, and stearic hindrance because of ODS and GPTMS.

In general, nano titania carry several –OH groups and pose the problem of agglomeration due to strong interaction between neighbouring titania particles due to van der Waal’s forces. Such agglomerates have to be broken by long time sonication to bring nanoparticles into good dispersion form so that their modification can be done. However, in this work, modification was done at the synthesis stage of nanoparticles, therefore extra steps for breaking the agglomerates were eliminated and particles with good dispersability were obtained. This is due to the presence of ODS and GPTS on the surface of nano titania which carry R-OH groups. In an aqueous medium, hydroxyl ions will be produced in the functional groups of silanes attached around the surface of nano titania. In simplified form, the modified nano titania will be surrounded by negatively charged species. As a result of similar charge repulsion combined with the stearic hindrance effect, deagglomerated particles are obtained as shown in Figure 7.

### 4.5. Reaction Schematics

**Figure 8:**
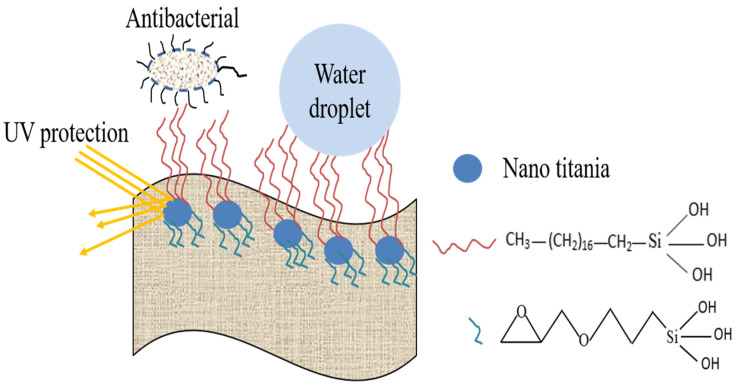
Fabric decorated with synthesized modified nano titania.

The schematic of the modification of cotton fabric with functionalized nano titania has been shown in Figure 6, where nano titania is attached to the fabric. While octadecyl trimethoxysilane as a hydrophobic agent is linked with titania nanoparticles thus providing a multifunctional fabric. In the reaction system, titanium isopropoxide, GPTMS, and ODS were present in ethanol at an acidic pH. Various reactions were expected to occur during the synthesis process of modified nano titania and the detailed mechanism is shown in Figure 8.

**Figure 9:**
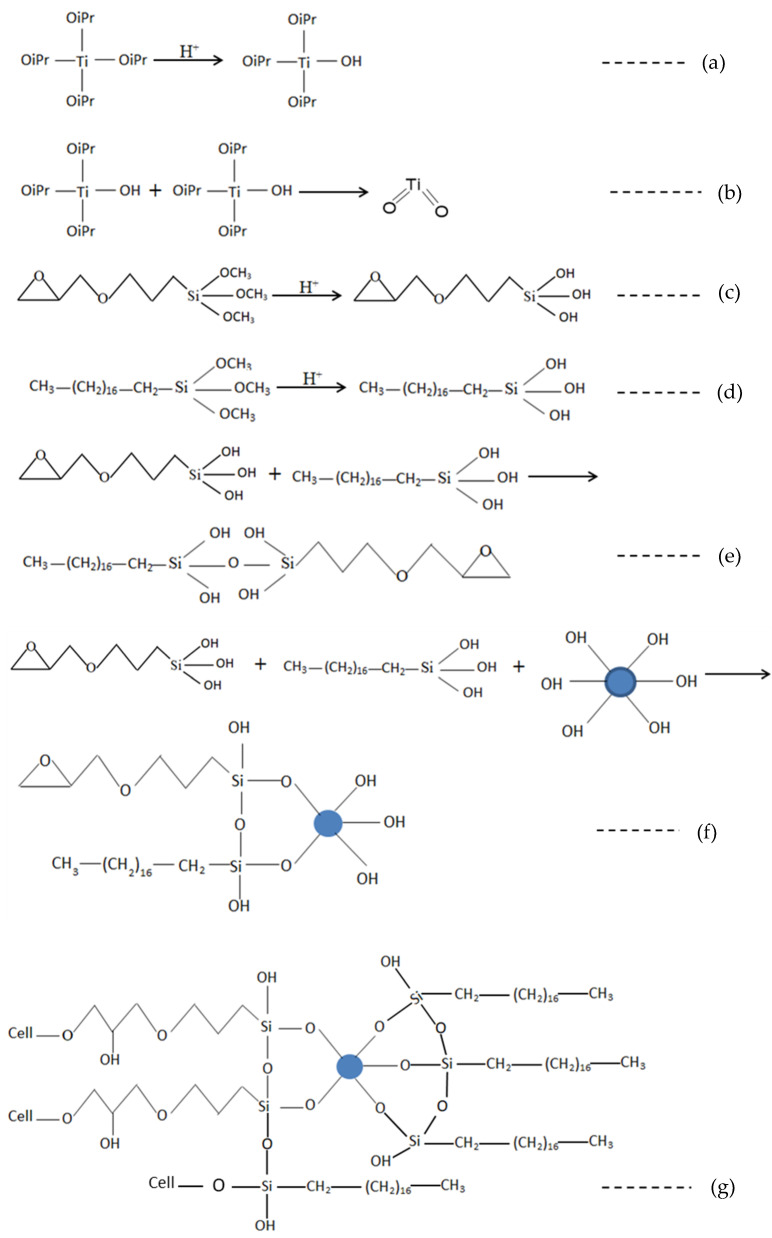
Various reactions expected during the process, (**a**) Hydrolysis of titanium isopropoxide. (**b**) Condensation reaction to form nano titania. (**c**) Hydrolysis of GPTMS. (**d**) Hydrolysis of ODS. (**e**) Condensation between hydrolysed GPTMS and ODS. (**f**) Synthesized modified nano titania with GPTMS and ODS. (**g**) Condensation between cellulose and synthesized modified nano titania.

Initially, nano titania was produced by hydrolysis and condensation of titanium isopropoxide (Figure 9a,b) carrying various -OH groups on their surface that can react further with silane functionalizing agents present in the system. Methoxy groups attached to -Si of silanes are hydrolyzable groups and produced silanol after hydrolysis (Figure 9c,d) that might undergo reaction with -OH groups present in the system; -OH of nano titania and that of hydrolysed silanes, (Figure 9e,f). When the synthesized dispersion was applied on cotton fabric, epoxy groups of GPTMS might undergo reaction with -OH of cellulose resulting in the anchoring of modified nano titania, while silanol groups of ODS moiety might have also reacted with the cellulose (Figure 9g). The silanol groups of organofunctional trialkoxysilanes can react with each other during the condensation reaction to form a continuous sol-gel matrix, but also with the hydroxyl groups on the surface of the cellulose fibres. The epoxy group was mainly available for reaction with the cellulose. Therefore, the predominant attachment of nano titania to the cellulose can be attributed to the epoxy group of GPTMS.

### 4.6. Hydrophobic Properties

**Figure 10:**
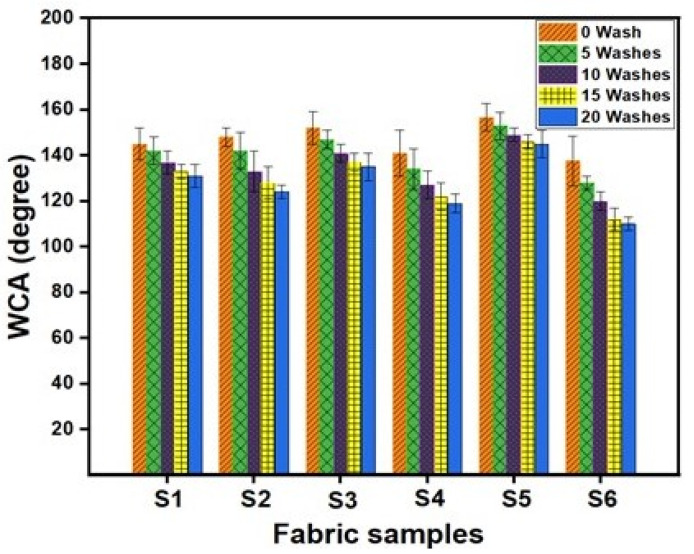
The water contact angle of modified fabrics before and after various washing cycles.

The water contact angle was measured to investigate the hydrophobic character of fabrics treated with various volumes of GPTMS and ODS in the presence of a fixed volume of TIP, and the results are presented in Figure 10. It can be noted that the surface of cotton fabrics, which were treated by the nano titania solution modified with 4% ODS and 6% GPTMS, showed a very high superhydrophobicity with a water contact angle of 156.69° (Sample S5). In contrast, the fabric treated with nano titania modified with ODS alone exhibited a water contact angle of 136°. For comparison, when water was dropped on the surface of pristine cotton fabric no contact angle could be observed and water was absorbed immediately by the fabric due to the abundant hydroxyl groups and numerous holes in the woven structure of textiles.

During their life, the treated fabrics go through various washing processes. To evaluate hydrophobic properties after washing process, the prepared fabric samples were washed, and the contact angle was measured. After the laundering process, the hydrophobicity of fabrics treated with modified nano titania could be reduced due to damaging the links formed by silanes and introducing filth such as detergent and moisture. The treated fabrics were subjected to various washing cycles to investigate the laundering durability of hydrophobic fabrics, and the results of contact angle after various washing cycles are depicted in Figure 10.

Fabric treated with nano titania modified with ODS alone exhibited a decrease in contact angle from 136° to 81° after five washing cycles. This indicated that the surface was slightly hydrophobic after five washing cycles, indicating the presence of very few hydrophobic moieties on the fabric surface, resulting in a significant decrease in the contact angle of the fabric. This decrease in contact angle can be ascribed to the removal of aggregates of nano titania from the fabric surface, leaving behind a slight hydrophobicity. On the contrary, fabrics treated with nano titania modified with GPTMS and ODS exhibited hydrophobic properties even after 20 washing cycles, as can be seen in Figure 8. Thanks to the epoxy group on GPTMS, which made it possible to firmly attach the hydrophobic moiety to cotton fibre with covalent bonding, no sharp decrease in contact angle was observed. Generally, textile surfaces exhibiting contact angles of >150° are considered super-hydrophobic and provide a lotus effect. Thus, before and after the washing process, the developed fabric samples have high contact angle values. As can be seen from Figure 10, sample S5 exhibited superhydrophobic properties after 10 washing cycles and maintained hydrophobic properties even after 20 washing cycles. Although a small decrease in contact angle was noted, that might be due to the removal of modified nano titania. GPTMS worked as a binding agent to attach nano titania and ODS to the fabric. Its higher volume, along with high levels of ODS, resulted in the generation of more nano roughness due to a higher level of chemical attachment of nano titania along with more attachment of ODS molecules that can be manifested by superhydrophobic fabrics that retained their superhydrophobicity after various washing cycles. This higher washing fastness is attributed to covalent bond formation between epoxy groups of GPTMS modified nano titania and hydroxyl groups of cotton. GPTMS, a silane crosslinker, formed a three-dimensional network, which improved the washing durability of the hydrophobic surface.

### 4.7. Water Floating Test

**Figure 11:**
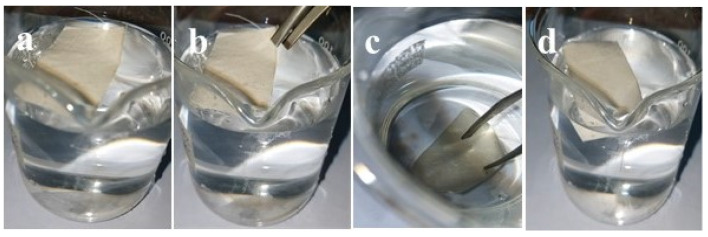
Water floating test of coated fabric with modified nano titania. (**a**) Coated fabric floating on the water surface. (**b**) Forcing down the fabric into water. (**c**) Coated fabric forced to the bottom of water filled beaker. (**d**) Fabric back on the water surface after removal of external force.

Water floating test of the fabric coated with modified nano titania further manifested the hydrophobic properties under pressure-initiated wetting. The pristine cotton fabric quickly absorbed water and sank to the bottom of the beaker. On the other hand, when fabric coated with modified nano titania was placed on the water surface, it kept on floating on the water surface (Figure 11a). When this fabric was submerged in water by an outer power (Figure 11b,c), the superhydrophobic cotton surfaces showed like a silver mirror, which is brought about by the trapped air layer on the superficial level of the coated fabric. Upon removal of external force, the fabric remained floating on the surface of the water without absorbing water (Figure 11d).

### 4.8. UV Protection Properties

**Figure 12:**
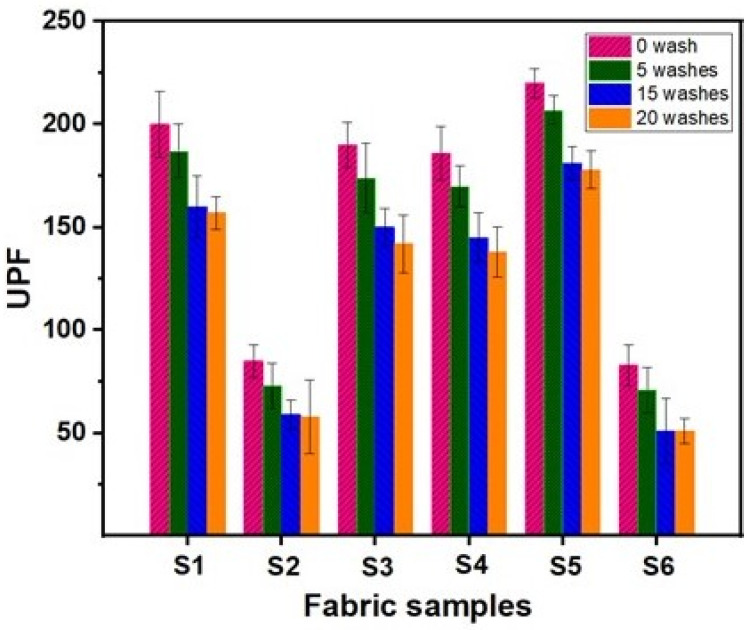
UPF of modified cotton fabrics before and after various washing cycles.

UV protection properties were quantified for cotton fabrics before and after treatment with functionalised nano titania and the results are shown in Figure 12. The UV-blocking capability of coated fabrics exhibiting superhydrophobicity was compared based on the UPF values. Each fabric sample was scanned five times for this purpose, and the average was recorded. Standard UPF protection rating in the range of 40–50 and >50 is considered excellent for fabrics, thereby providing effective protection against harmful UV radiation [45]. The UPF value of the pristine cotton fabric was 6, and the values for the fabric treated with functionalised TiO_2_ nanoparticles exhibited higher UPF than untreated fabrics due to the ability of nano titania to block UV rays by absorption and scattering. This increase in UPF rating has been attributed to a decrease in the interstices of the fabric due to the covering of yarns by modified nano titania coating. It is observed that the sample S6 shows the minimum value of UPF corresponding to the least amount of GPTMS applied, while S5 shows the maximum UPF value due to the higher amount of crosslinker (GPTMS) used during application. This result indicated that with an increasing amount of GPTMS, more nano titania gets attached to the fabric surface. The UPF value of 220 in sample S5 is attributed to the covalent bond formation between hydroxyl groups of cotton and epoxy groups of GPTMS, which renders the highest UPF. An obvious decrease in UPF was observed after various washing cycles that might be due to the removal of the top layer of modified nano titania. However, the layer of modified nano titania that was directly attached to the cellulosic chain resists further laundering cycles, maintaining an appreciable UV protection rating after 15–20 washing cycles (Figure 12).

### 4.9. Antibacterial Properties

**Figure 13:**
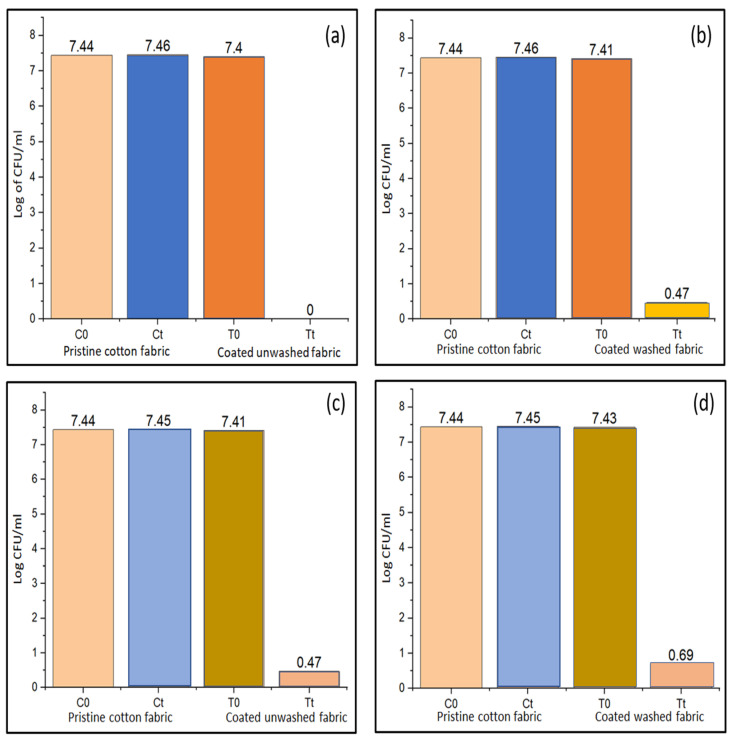
Antibacterial properties of (**a**) the pristine cotton fabric and coated unwashed cotton fabric against *S. aureus*, (**b**) the pristine cotton fabric and coated washed fabric after 20 washing cycles against *S. aureus*, (**c**) the pristine cotton fabric and coated unwashed cotton fabric against *E. coli*, (**d**) the pristine cotton fabric and coated washed fabric after 20 washing cycles against *E. coli*.

Biological self-cleaning of coated fabric was studied against *S. aureus* and *E. coli* as these are common invading bacteria that affect human health and belong to the class of Gram-positive and Gram-negative bacterial strains, respectively. Modified sample S5 was selected for evaluation of antibacterial properties as it exhibited the highest UPF value, indicating higher attachment of modified nano titania. Quantitative antibacterial properties of the coated fabric were estimated and results are presented in Figure 13, for the bacterial cell count at *t* = 0 (just after fabric contamination with bacteria) and at *t* = 24 h (after 24 h of incubation period), indicating the change in bacterial growth with time. As can be seen in Figure 13a–d, an increase in cell count was found from 7.44 log CFU mL^−1^ to 7.46 log CFU mL^−1^ and from 7.44 log CFU ml^−1^ to 7.45 log CFU mL^−1^, in the case of pristine cotton fabric after its contamination with *S. aureus* and *E. coli*, respectively. It demonstrated that the pristine cotton fabric was not resistant to bacteria and would get damaged by the deteriorating actions of bacterial attack. When modified nano titania coated fabric was exposed to bacterial strains of *S. aureus* and *E. coli*, a reduction in cell count was noted after 24 h of the incubation period. The unwashed fabric exhibited *S. aureus* count reduction from 7.1 log CFU mL^−1^ at *t* = 0 to 0 log CFU mL^−1^ at *t* = 24 h (Figure 13a). The same coated fabric after 20 washing cycles showed an *S. aureus* count reduction from 7.41 log CFU mL^−1^ to 0.47 log CFU mL^−1^ (Figure 13b). This decrease in bacterial count exhibited the bacterial resistant properties of the coated fabric before wash and after 20 washing cycles.

When the same coated fabric was tested for its antibacterial properties against *E. coli*, a reduction in cell growth was noted from 7.41 log CFU mL^−1^ to 0.47 log CFU mL^−1^ (Figure 13c) and from 7.43 log CFU mL^−1^ to 0.69 log CFU mL^−1^ (Figure 13d) for unwashed coated fabric and after 20 washing cycles of the same coated fabric, respectively. The reduction in bacterial growth could be due to the generation of ROS (reactive oxygen species) from modified nano titania present on the fabric surface. Moreover, the obtained cell count reduction indicated that the antibacterial activity of the coated cotton fabric is higher against *S. aureus* than *E. coli*. The percentage of bacterial decline for coated unwashed fabric against *S. aureus* and *E. coli* was noted to be 99.99% and 93%, respectively. Coated fabric after being subjected to 20 laundering cycles demonstrated a percentage mortality of *S. aureus* and *E. coli* of 93% and 90%, respectively. This decrease in percentage of cell growth can be attributed to the removal of some of the modified nano titania from the fabric surface due to mechanical actions and the hot aqueous detergent solution that the fabric was subjected to during the various laundering cycles. Despite the removal of a few of the modified nano titania from the coated fabric, the coated fabric still maintained its antibacterial properties against both strains of bacteria. This manifested good adhesion of modified nano titania on the fabric surface and sustained antibacterial properties after various laundering cycles.

### 4.10. Surface Roughness

To mimic lotus surface properties on fabric, surface roughness and low-energy chemicals are usually incorporated into textile surfaces. After the application of modified nano titania, the surface topography of the fabric alters as discussed earlier. To analyse the surface properties of the fabric treated with modified nano titania, the Kawabata evaluation system was used to measure the surface properties of the pristine fabric and coated fabric, and the results are presented in Table 2. On investigation of the surface properties of both fabrics, the frictional parameters MIU, which is the coefficient of friction, and MMD, which is frictional roughness, were greater for coated cotton fabric than for pristine cotton fabric. These frictional parameters rely on the fibre’s contact area. After coating the fabric surface with modified nano titania, surface friction was increased. Moreover, the surface roughness of the coated fabric was also increased, as indicated by the increased value of SMD, which is geometrical roughness. The presence of nanoparticles on the fabric surface caused a difference in the thin and thick places of the coated fabric surface, enhancing the roughness of the surface.

### 4.11. Air Permeability

Air permeability of the fabric is an important factor that plays a significant role in determining fabric comfort properties. Pristine cotton fabric and coated fabric were tested for their air permeability. For pristine cotton fabric, air permeability was 548.75 mm^3^/s, which decreased to 436.5 mm^3^/s after the application of modified nano titania. Embedding nanoparticles and cross-linking network of silanes caused some filling of the fabric porous structure, which resulted in a decrement of air permeability of the fabric, but this decrease was not significant and could be ignored.

### 4.12. Tear Strength

**Figure 14:**
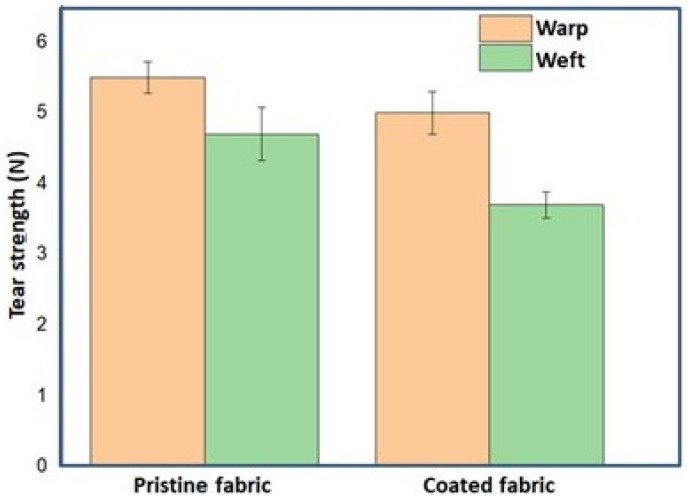
Tear strength of pristine fabric and fabric treated with modified nano titania.

To analyse the effect of the modified nano titania on the mechanical characteristics of treated cotton, the tear strength of the fabric treated with modified nano titania was determined and compared with the tear strength of the pristine fabric. As can be seen from Figure 14, the tear strength of treated fabric decreased due to the cross-linking effect of GPTMS that restricted yarn slippage upon application of force. However, this decrease in tear strength can be ignored due to its insignificant change, demonstrating that the coating has not much affected the mechanical characteristics of the treated fabrics.

## 5. Conclusions

In conclusion, the robust multifunctional cotton fabric was fabricated via facile synthesis of modified hydrophobic nano titania in one bath. Prepared modified nano titania were of anatase form, having a size of 17.9 nm as confirmed by XRD. The zeta potential (−23.3 mV) indicated their good dispersibility. The presence of GPTMS on the surface of nano titania was responsible for enhanced durability of functional properties like UV protection, antibacterial activity, and physical self-cleaning, mimicking the self-cleaning properties of the lotus leaf as determined after application on a cellulosic substrate. Due to the presence of epoxy and siloxane groups, the prepared finish can be applied to substrates having amide, amine, and hydroxyl groups under suitable conditions. For UV protection, a maximum of 220 UPF values was achieved and, after various washing cycles, excellent UV protection was sustained. The coated substrate manifested a percentage bacterial reduction of 99.99% and 93% for Gram-positive and Gram-negative bacterial strains, respectively. Furthermore, the presence of ODS on the surface of nano titania resulted in the progress of superhydrophobic fabric showing a 157.9° contact angle. All the results showed the development of a multifunctional cellulosic substrate having UV protection, antibacterial, and superhydrophobicity with ameliorating durability. The developed approach is simple, less time-consuming, and feasible for industrial fabrication.

## Figures and Tables

**Table 1:** Diverse amounts of GPTMS and ODS used to develop coated fabrics.

Sample	GPTMS (%)	ODS (%)
Control	0	2
S1	6	2
S2	2	4
S3	4	4
S4	4	2
S5	6	4
S6	2	2

**Table 2:** Surface properties as determined by the Kawabata evaluation system.

	MIU	MMD	SMD
Pristine fabric	0.182	0.0137	3.85
Coated fabric	0.374	0.02	5.206

## Data Availability

All the data is presented within the manuscript.
